# Natural Antisense Transcript PEBP1P3 Regulates the RNA Expression, DNA Methylation and Histone Modification of *CD45* Gene

**DOI:** 10.3390/genes12050759

**Published:** 2021-05-17

**Authors:** Zhongjing Su, Guangyu Liu, Bin Zhang, Ze Lin, Dongyang Huang

**Affiliations:** 1 Department of Histology and Embryology, Shantou University Medical College, No. 22, Xinling Road, Shantou 515041, China; 18bzhang@stu.edu.cn; 2 Department of Cell Biology, Shantou University Medical College, No. 22, Xinling Road, Shantou 515041, China; 16gliu@stu.edu.cn; 3 Department of Central Laboratory, Shantou University Medical College, No. 22, Xinling Road, Shantou 515041, China; zelin@stu.edu.cn

**Keywords:** CD45, natural antisense transcript, DNA methylation, histone modification

## Abstract

The leukocyte common antigen CD45 is a transmembrane phosphatase expressed on all nucleated hemopoietic cells, and the expression levels of its splicing isoforms are closely related to the development and function of lymphocytes. PEBP1P3 is a natural antisense transcript from the opposite strand of *CD45* intron 2 and is predicted to be a noncoding RNA. The genotype-tissue expression and quantitative PCR data suggested that PEBP1P3 might be involved in the regulation of expression of CD45 splicing isoforms. To explore the regulatory mechanism of PEBP1P3 in CD45 expression, DNA methylation and histone modification were detected by bisulfate sequencing PCR and chromatin immunoprecipitation assays, respectively. The results showed that after the antisense RNA PEBP1P3 was knocked down by RNA interference, the DNA methylation of *CD45* intron 2 was decreased and histone H3K9 and H3K36 trimethylation at the alternative splicing exons of *CD45* DNA was increased. Knockdown of PEBP1P3 also increased the binding levels of chromatin conformation organizer CTCF at intron 2 and the alternative splicing exons of *CD45*. The present results indicate that the natural antisense RNA PEBP1P3 regulated the alternative splicing of CD45 RNA, and that might be correlated with the regulation of histone modification and DNA methylation.

## 1. Introduction

CD45, also known as protein tyrosine phosphatase receptor type C (PTPRC), is mainly expressed on immune cells to initiate cross-membrane signal transduction [1,2]. The primary transcript of CD45 uses three alternative exons to produce a variety of RNA and protein isoforms, CD45 RA (including exon 4), RB (including exon 5), RC (including exon 6) and RO (excluding exons 4-5-6). Distinct CD45 isoforms are expressed on lymphocyte subsets at different developmental stages and regulate different functional statuses of lymphocytes [3,4]. In the clinic, the abnormal expression of CD45 isoforms is correlated with the pathogenesis of some autoimmune or immune deficiency diseases [5].

Regarding the regulation of CD45 splicing, previous studies found that nucleotide polymorphisms at the splicing site of CD45 alternative exons influenced the expression of CD45 isoforms and the function of immune cells [6,7,8], and heterogeneous ribonucleoprotein hnRNPs were also identified as critical regulators of CD45 alternative splicing [9,10,11]. In addition, DNA hypermethylation could promote the inclusion of CD45 exon 5 by mediating local RNA polymerase II pausing and the transcriptional elongation speed of pre-mRNA [12,13,14], which indicates that the alternative splicing of CD45 RNA is regulated at the co-transcriptional level.

*PEBP1P3* (*phosphatidylethanolamine binding protein 1 pseudogene 3*) is a natural antisense gene of *CD45* and annotated as a pseudogene in the human genome. Through epigenetic and gene structure analysis, we previously issued the viewpoint that the transcript of PEBP1P3 might be involved in the expressional regulation of CD45 isoforms in autoimmune diseases [15]. To investigate the regulatory effect of PEBP1P3 on the expression of CD45 in this study, we first analyzed the correlation between the RNA levels of CD45 and PEBP1P3 in different human normal tissues from genotype-tissue expression (GTEx). Then, the antisense transcript PEBP1P3 in cultured lymphocytes was knocked down by RNA interference (RNAi), and reverse transcription and quantitative PCR analysis (RT-qPCR) were applied to detect the expression of CD45 RNA isoforms. Bisulfate sequencing PCR, chromatin immunoprecipitation (ChIP) and bioinformatics prediction were also applied to explore the possible regulatory mechanism of PEBP1P3 in CD45.

## 2. Materials and Methods

### 2.1. Database and Software

Genomic and cDNA sequences of *CD45* and its antisense gene *PEBP1P3* were obtained from GenBank (available online: http://www.ncbi.nlm.nih.gov, accessed on 9 August 2018) and UCSC Genome Browser (available online: http://www.genome.ucsc.edu, accessed on 9 August 2018). Their coding potential was analyzed using the online software Coding Potential Calculator (CPC) (available online: http://cpc.gao-lab.org/, accessed on 1 May 2021) [16]. The levels of CD45 and PEBP1P3 RNA were extracted from GTEx (available online: https://www.gtexportal.org, accessed on 9 August 2018), which is a project creating and releasing RNA expression data from 8555 tissue samples obtained from 570 adult postmortem individuals [17]. The motif of CTCF binding sites within *CD45* DNA was predicted through CTCFBSDB 2.0 (available online: http://insulatordb.uthsc.edu, accessed on 1 May 2021) [18,19].

### 2.2. Cell Culture

HuT 78 and Jurkat human T lymphocytes and RPMI 8226 human B lymphocytes were obtained from the Stem Cell Bank, Chinese Academy of Sciences. HuT 78 cells were cultured in IMDM (Sigma-Aldrich, St. Louis, MO, USA) with 20% fetal bovine serum (Invitrogen, Carlsbad, CA, USA). Jurkat and RPMI 8226 cells were cultured in RPMI 1640 medium (Sigma-Aldrich) with 10% fetal bovine serum. The cells were incubated at 37 °C with 5% CO_2_.

### 2.3. RNAi Assay

The siRNA oligonucleotides targeting PEBP1P3 (si-PEBP1P3 sense 5′-GUGAUCCUAAUACCAGAAUTT-3′ and si-PEBP1P3 antisense 5′-AUUCUGGUAUUAGGAUCACTT-3′) and negative control (si-NC sense5′-UUCUCCGAACGUGUCACGUTT-3′ and si-NC antisense 5′-ACGUGACACGUUCGGAGAATT-3′) were designed and synthesized by Genepharma (Shanghai, China). Synthetic RNAi was transfected into Jurkat, HuT 78 and RPMI 8226 cells, respectively, with Lipofectamine RNAiMAX Reagent (Invitrogen) for 48 h based on the manufacturer’s instructions.

### 2.4. RNA Extraction and RT-qPCR

Total RNA of the cell lines was extracted with TRIzol^®^ reagent (Invitrogen) according to the manufacturer’s protocol. To detect the subcellular localization of the transcripts, the cytoplasmic and nuclear RNA fractions were prepared with a RNeasy Mini kit (QIAGEN, Hilden, Germany). RNA quality was examined by the ratio of OD260/OD280 and RNA electrophoresis, and then the RNAs were reverse-transcribed with a QuantiTect Reverse Transcription kit (QIAGEN) for genomic DNA removal and cDNA synthesis. The qPCR reactions were performed using SYBR Green qPCR SuperMix-UDG (Invitrogen) in an Applied Biosystems PRISM 7500 (Thermo Fisher, Waltham, USA). All the qPCR primers used are listed in Table 1. GAPDH was used as an internal reference to calculate the expression of target RNA, and U6 spliceosomal small nuclear RNA (snRNA) was used as a marker of nuclear RNA.

### 2.5. Bisulfite Sequencing PCR for DNA Methylation Analysis

The genomic DNA of treated cells was prepared using a DNA extraction kit and subsequently treated with the DNA Bisulfite Conversion Kit (QIAGEN) according to the manufacturer’s instructions. For detecting DNA methylation of CD45 intragenic CpG sites, bisulfite sequencing PCR primers (Bis-I2F 5′-TTAGGTTGGAGTGTAGTGGTTT-3′ and Bis-I2R 5′-CCCATCCTCAACAATAAATCTA-3′ for intron 2; Bis-E5F 5′-TAGTGGGGGAAGATTGATGTA-3′ and Bis-E5R 5′-AATTAAATTTCCTCACACCATTCT-3′ for exon 5) were designed according to the bisulfite-treated DNA sequences. After amplification, PCR products were purified with a Universal DNA Purification Kit (Tiangen, Beijing, China) and cloned into the pGM-T vector. The inserted PCR fragments from individual clones were sequenced to enable determination of the status of CpG sites.

### 2.6. Chromatin Immunoprecipitation

ChIP assays were performed with EZ-Magna ChIP A Kits (Merck Millipore, Billerica, USA) according to the manufacturer’s protocol. ChIP grade antibodies were used in the present study as follows: anti-histone H3 lysine 4 trimethyl (H3K4me3, Abcam, ab8580), anti-histone H3 lysine 9 trimethyl (H3K9me3, Invitrogen, 49–1008), anti-histone H3 lysine 36 trimethyl (H3K36me3, Abcam, ab9050), anti-H3acetyl (Abcam, ab47915), anti-histone H3 lysine 9 (H3K9ac, Invitrogen, 49–1009), anti-CTCF (Invitrogen, PA5–46847) and normal rabbit IgG. Immunoprecipitated DNA was analyzed by qPCR and normalized to the input DNA, and each experiment was repeated three times independently. The sequences of the primers in reference to the different regions of the CD45 gene are listed in Table 1.

## 3. Results

### 3.1. The Expression of CD45 RNA Was Positively Correlated with the Level of PEBP1P3 RNA in Multiple Human Tissues

**Figure 1:**
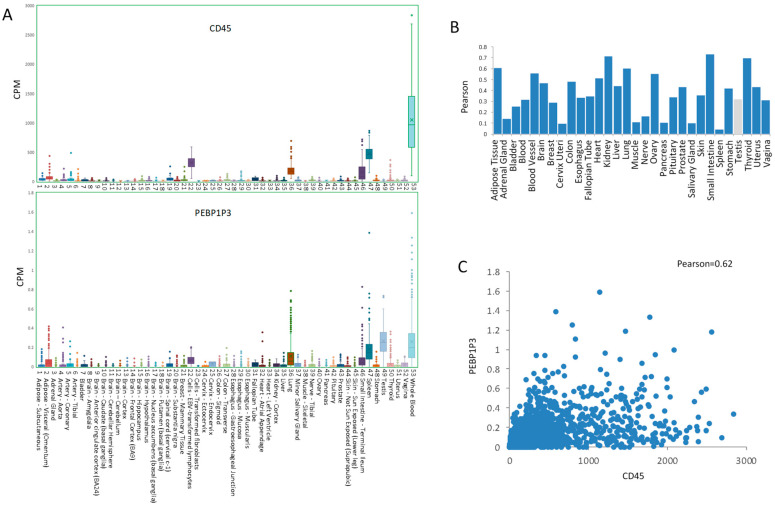
Expression of CD45 and PEBP1P3 in different tissues and cells of humans. (**A**) The RNA levels of CD45 and PEBP1P3 in 53 specific tissues from GTEx RNA-seq of 8555 samples from 570 donors. (**B**) The Pearson correlation between CD45 and PEBP1P3. Dark blue indicates a positive correlation in most detected tissues, and light gray indicates a negative correlation in the testis. (**C**) The value distribution of individual samples and the Pearson correlation (0.62) between CD45 and PEBP1P3 for the total samples except testis.

Based on RNA-seq data from the GTEx from 51 specific tissues and two cell lines, the expression level of CD45 from high to low was whole blood > spleen > EBV-transformed lymphocytes > lung > small intestine > adipose > other types of tissues. Whole blood had the highest expression level, with a median expression of 969.96 CPM. For PEBP1P3, the expression level was testis > whole blood > spleen > lung > small intestine > EBV-transformed lymphocytes > other types of tissues (Figure 1A). The highest expression of PEBP1P3 among these samples was 0.25 CPM in the testis. The expression levels of CD45 in multiple human tissues were positively correlated with that of PEBP1P3, and the total Pearson correlation was 0.62, except for the testis (Figure 1B,C).

### 3.2. The Expression and Subcellular Distribution of PEBP1P3 in Lymphocytes

**Figure 2:**
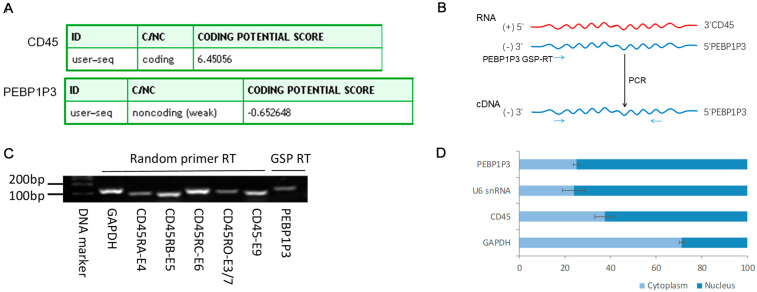
The coding potential and subcellular distribution of CD45 and PEBP1P3 RNA. (**A**) The coding potential predicted with the online web server CPC. (**B**) Identification of the CD45 antisense RNA PEBP1P3 in HuT 78 cells through gene-specific primer (GSP) RT-PCR and sequencing. (**C**) RT-PCR and agarose gel electrophoresis of CD45 isoforms and PEBP1P3. (**D**) The subcellular distribution ratio of RNA determined by RT-qPCR after the separation of the nuclear and cytoplasmic fractions in HuT 78 cells.

The full-length CD45 RNA sequence recorded at GenBank was 5429 bp and composed of 33 exons, and PEBP1P3 RNA was 1452 bp and composed of two exons. Analysis of the RNA sequences with the online web server CPC predicted that the coding scores for CD45 and PEBP1P3 were 6.45 and −0.65, respectively, which indicated that the PEBP1P3 transcript was a noncoding RNA (Figure 2A). We applied gene-specific primer RT-PCR and sequencing to detect the expression of PEBP1P3, and the results identified that the expression of PEBP1P3 was from the antisense strand of CD45 in cultured HuT 78 lymphocytes, and the RNA level of PEBP1P3 was lower than that of CD45 (Figure 2B,C). The nuclear and cytoplasmic RNA separation and RT-qPCR results showed that PEBP1P3 was distributed more in the nucleus (74.89%) than in the cytoplasm (25.11%), and that ratio was similar to the reference nuclear U6 snRNA (Figure 2D).

### 3.3. Knockdown of PEBP1P3 Altered the Expression of CD45 Isoforms

**Figure 3:**
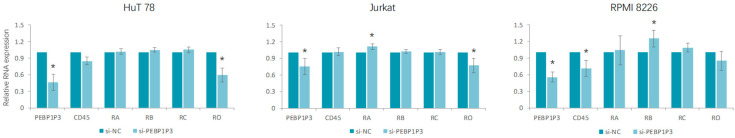
The effect of PEBP1P3 knockdown on the RNA expression of CD45. The expression levels of CD45 RNA were checked after knockdown of the antisense transcript PEBP1P3 through RNAi in HuT78, Jurkat and RPMI 8226 cells, respectively (si-NC, interference of negative control; si-PEBP1P3, interference of PEBP1P3). The values of CD45 and PEBP1P3 were normalized to GAPDH, and CD45 isoforms were normalized to constant CD45. All data are presented as mean ± standard error of the mean (SEM) from three independent experiments. * *p* < 0.05.

After transfection of siRNA targeting PEBP1P3 in HuT 78, Jurkat and RPMI 8226 cells for 48 h, PEBP1P3 RNA decreased in the interfered cells, and CD45 RNA (based on the expression of constitutive exon 9) decreased in the RPMI 8226 cell line (*p* < 0.05). Knockdown of PEBP1P3 also resulted in reduced RNA levels of the CD45RO isoform (*p* < 0.05 in HuT 78 and Jurkat cells, *p* > 0.05 in RPMI 8226 cells), increased CD45RA in Jurkat cells and increased CD45RB in RPMI 8226 cells (Figure 3).

### 3.4. PEBP1P3 RNA Regulated DNA and Histone Modification of CD45

**Figure 4:**
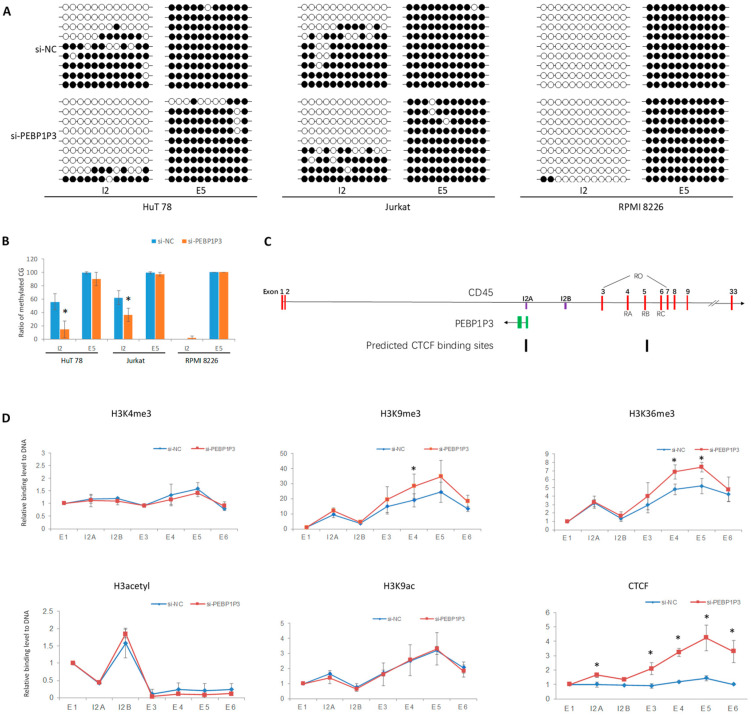
The effect of PEBP1P3 knockdown on DNA and histone modification of CD45. (**A**,**B**) The DNA methylation status and methylation ratio of CpG sites on CD45 intron 2 (I2) and exon 5 (E5) after PEBP1P3 was knocked down in HuT 78, Jurkat and RPMI 8226 cells. Closed circles represent methylated CpG sites, and open circles represent unmethylated CpG sites. (**C**) The sketch indicates the locational relationship of the *CD45* and *PEBP1P3* genes, CD45 RNA isoforms, locations of primers applied in ChIP and two potential CTCF binding sites predicted through online CTCFBSDB 2.0. (**D**) The levels of trimethylated histone H3K4 (H3K4me3), trimethylated histone H3K9 (H3K9me3), trimethylated histone H3K36 (H3K36me3), acetylated histone (H3acetyl), acetylated H3K9 (H3K9ac) and CTCF binding at different locations of *CD45* DNA after PEBP1P3 was knocked down in HuT 78 cells. Each experiment was repeated three times, and the quantification was presented as the mean ± SEM. si-NC: interference of negative control; si-PEBP1P3, interference of PEBP1P3; * *p* < 0.05.

After PEBP1P3 RNA was knocked down by RNAi, DNA at intron 2 of *CD45* was hypomethylated. There was no significantly changed ratio of DNA methylation at exon 5 of *CD45*, where the CpG sites maintained a highly methylated status (Figure 4A,B). According to the genomic segments broken by ultrasonication in ChIP, several pairs of primers were designed to detect the modified histones or factors binding to *CD45* DNA in HuT 78 cells (Figure 4C). ChIP and qPCR detected a peak level of acetylated H3 at the intron 2B site of *CD45* in HuT 78 and peaks of H3K9ac, H3K9me3, H3K36me3 and CTCF at intron 2A and exon 5. After RNAi of PEBP1P3, the peaks of H3K9me3 and H3K36me3 at exon 4–5 increased (*p* < 0.05). For CTCF, the peaks at intron 2 and exon 5 increased after PEBP1P3 knockdown (*p* < 0.05) (Figure 4D).

## 4. Discussion

Natural antisense transcripts are RNA sequences transcribed from opposite DNA strands at the same locus of the sense gene [20,21], and different antisense genes have different features of coding potential and regulatory effects on the sense gene [22,23,24]. The GTEx data indicated that CD45 and its natural antisense RNA PEBP1P3 were highly expressed in lymphoid-associated tissues, and the RNA levels of PEBP1P3 were positively correlated with the expression of CD45 in multiple human tissues except for the testis. To detect whether there was a regulatory effect of PEBP1P3 on the expression of the sense gene *CD45*, we knocked down PEBP1P3 in cultured lymphocytes by RNAi. The RT-qPCR results showed that the knockdown of PEBP1P3 decreased the expression of CD45 in human cell lines of HuT 78 and RPMI 8226. The RNA levels of PEBP1P3 and CD45 in cultured lymphocytes HuT 78 and RPMI 8226 were consistent with their expression in human tissues based on the GTEx data. In addition to the effect on CD45 RNA, the RNAi results also indicated the regulatory effect of PEBP1P3 on the expression of CD45 alternative splicing isoforms, mainly to increase the expression of CD45 RB and decrease the expression of CD45 RO. Since CD45 isoforms indicate the development of lymphocytes and the activity of the immune system [3,4,5], the present study also provides clues for studying immune activity in clinically associated diseases.

The noncoding RNA PEBP1P3 had a relatively low expression level compared to CD45 and mainly existed in the nucleus, where the transcription and splicing of RNA occurs. A previous study indicated that DNA methylation and histone modification regulated the alternative splicing of weak exons in pre-mRNA at co-transcriptional level through controlling the RNA polymerase II transcription elongation rate. Methylated DNA sequences are correlated with fast transcriptional rate and skipping of the exons, and unmethylated DNA sequences are correlated with slow transcriptional rate and the enough time for spliceosomes to recognize each exon of the primary transcript [13,14,25]. Splicing of CD45 pre-RNA occurred during the transcriptional process, and exons 4, 5 and 6 were relatively weak splicing sites [1,26,27]. Based on analyzing the feature of the non-coding RNA PEBP1P3, we proposed that PEBP1P3 RNA might recruit DNA methyltransferase and histone modification modulators specifically targeting *CD45* DNA and contribute to the regulation of the transcriptional rate and the splicing of the alternative exons [15].

To explore the possible mechanism of PEBP1P3 in the splicing regulation of CD45, we detected the DNA methylation of *CD45* after PEBP1P3 RNA was knocked down in the cell line of HuT 78, since HuT 78 cells manifested the best knockdown efficiency among the three PEBP1P3 RNAi cell lines as shown in Figure 3. The results indicated that the antisense transcript PEBP1P3 regulated DNA methylation at intron 2 of CD45, which is exactly the gene locus of the oppositely transcribed PEPB1B3. The DNA methylation status at the alternative exon 5 of CD45 did not show obvious alteration. In addition to DNA methylation, histone modifications have also been reported to be associated with transcriptional regulation [28,29]. Generally, transcriptionally active chromatin is associated with histone hyperacetylation and histone methylation of H3K4 and H3K36. Conversely, transcriptional repression is commonly associated with histone hypoacetylation and histone methylation of H3K9 and H3K27 [30]. Moreover, histone H3 acetylation, histone H3K4me3, H3K9me3 and H3K36me3 are also correlated with the splicing of alternative exons [31,32,33,34]. Given that the expressional regulatory effects of PEBP1P3, we detected the histone acetyl, H3K9ac, H3K36me3 and H3K4me3 by ChIP after the knockdown of PEBP1P3 RNA in HuT 78 cells. Acetylated histones manifested a binding peak at intron 2B of CD45 but did not show obvious changes after knocking down PEBP1P3. One possibility for this is that there were no changes. Another possibility is that the antibodies against acetylated histones we applied in ChIP were common to acetyl K9 + K14 + K18 + K23 + K27 of H3, and the different regulatory effects of these sites might mask some specific sites of acetylated histones. To identify the effect on exact lysine sites of histone H3, the presentative H3K9ac were detected by ChIP, and the results did not show significant difference after the knockdown of PEBP1P3. For histone methylation, H3K4me3 did not show peaks on *CD45* DNA in the si-NC control or si-PEBP1P3 knockdown HuT 78 cells. H3K9me3 and H3K36me3 presented elevated levels at exon 4–5 of *CD45*. H3K36me3 is generally enriched at transcribed regions of genes, and it is believed to modulate exon definition by regulating the rate of RNA polymerase II elongation and alternative splicing [30,32,35]. H3K9me3 was also reported as a feature of the alternative exons, and linked with the elongation of transcription [36,37,38]. The DNA methylation and histone modification results indicated that PEBP1P3 might regulate the alternative splicing of CD45 RNA through DNA methylation of intron 2 and histone modification of alternative exons.

Among the weakly spliced exons 4, 5 and 6 of CD45, splicing out of exon 5 is the key step for the formation of CD45RO [1,26,27], while intron 2 of CD45 is the location opposite to PEBP1P3. The question is how PEBP1P3, in contrast to intron 2, regulates the splicing of CD45 exon 5. Is there chromatin conformation alteration correlated with this function? In the human genome, the three-dimensional organization of chromatin is highly regulated and likely to play important roles in the regulation of gene expression [39,40,41]. The most common situation identified by previous studies was the loop between the promoter and enhancer, which resulted in long-distance regulation, and the key factor for regulating the chromatin architecture is CTCF [42,43]. The online software CTCFBSDB predicted that there were CTCF binding sites on the location of intron 2 and exon 5 of *CD45* DNA as in Figure 4C, and the ChIP results indicated that the knockdown of PEBP1P3 resulted in an increased binding level of CTCF on the peak of exon 5 and intron 2. Previous research identified that DNA methylation within the coding sequence could inhibit the binding of CTCF [12,44,45]. Based on these results, we proposed that PEBP1P3 might regulate the DNA methylation of CD45 on opposite strands, influence the binding of CTCF and the formation of a DNA loop between intron 2 and exon 5 of CD45, and finally regulate the splicing of exons through histone modification.

## Figures and Tables

**Table 1:** qPCR primers for detecting CD45 and PEBP1P3 RNA or DNA.

Name	Sequence (5′-3′)	Usage
CD45 E1-F	AGTAAAACCGAATCTGACATCATCACC	CD45 E1 DNA
CD45 E1-R	TGTTGTCTTATCAGACGAGGAACAATT	
CD45-I2A-F	TGCTTTGCTCTATTACTGTGGGC	CD45 I2A DNA
CD45-I2A-R	ATATTCTGGTACTGTGATGGGGTCA	
CD45-I2B-F	GCTATGGTGCCACCTACTGAAA	CD45 I2B DNA
CD45-I2B-R	ACAGTTACTACATTCTACACTTTGACT	
CD45 E3-F	TTGCCACTTGGTGAATGTTCTATC	CD45 E3 DNA
CD45 E3-R	GGAAGGTGTTGGGCTTTGC	
CD45 E4-F	GCAAAGATGCCCAGTGTTCCACTT	CD45 RA/E4 DNA
CD45 E4-R	TTCTCTTTCAAAGGTGCTTGCGG	
CD45 E5-F	TCATCAGTACAGACGCCTCACCTT	CD4 RB/E5 DNA
CD45 E5-R	CTGAATGTCTGCGTGTCAGTTCCA	
CD45 E6-F	AGCACCTTTCCTACAGACCCAGTT	CD45 RC/E6 DNA
CD45 E6-R	TGTTCGCTGTGATGGTGGTGTT	
CD45 E3/7-F	ACCTTCCCCCACTGATGCCTA	CD45 RO
CD45 E7-R	GTGGTTGAAATGACAGCGCTTC	
CD45 E9-F	GCACAAACAATGAGGTGCATAACC	CD45 RNA
CD45 E9-R	ATGTCTTATCAGGAGCAGTACATGA	
PEBP1P3-F	ATTCTGGTACTGTGATGGGGTCA	PEBP1P3 RNA
PEBP1P3-R	GATGCTTTGCTCTATTACTGTGGG	
GAPDH-F	GCACCGTCAAGGCTGAGAAC	GAPDH RNA
GAPDH-R	TGGTGAAGACGCCAGTGGA	
U6-F	GCTTCGGCAGCACATATACTAAAAT	U6 snRNA
U6-R	CGCTTCACGAATTTGCGTGTCAT	

## References

[1] Hermiston M.L., Xu Z., Weiss A. (2003). CD45: A critical regulator of signaling thresholds in immune cells. Annu. Rev. Immunol..

[2] Chang V.T., Fernandes R.A., Ganzinger K.A., Lee S.F., Siebold C., McColl J., Jonsson P., Palayret M., Harlos K., Coles C.H. (2016). Initiation of T cell signaling by CD45 segregation at ‘close contacts’. Nat. Immunol..

[3] Zikherman J., Weiss A. (2008). Alternative splicing of CD45: The tip of the iceberg. Immunity.

[4] Courtney A.H., Shvets A.A., Lu W., Griffante G., Mollenauer M., Horkova V., Lo W.L., Yu S., Stepanek O., Chakraborty A.K. (2019). CD45 functions as a signaling gatekeeper in T cells. Sci. Signal..

[5] Rheinlander A., Schraven B., Bommhardt U. (2018). CD45 in human physiology and clinical medicine. Immunol. Lett..

[6] Boxall S., Stanton T., Hirai K., Ward V., Yasui T., Tahara H., Tamori A., Nishiguchi S., Shiomi S., Ishiko O. (2004). Disease associations and altered immune function in CD45 138G variant carriers. Hum. Mol. Genet..

[7] Windhagen A., Sonmez D., Hornig-Do H.T., Kalinowsky A., Schwinzer R. (2007). Altered CD45 isoform expression in C77G carriers influences cytokine responsiveness and adhesion properties of T cells. Clin. Exp. Immunol..

[8] Samaan S., Guerin-El Khourouj V., Auboeuf D., Peltier L., Pedron B., Ouachee-Chardin M., Gourgouillon N., Baruchel A., Dalle J.H., Sterkers G. (2011). Outcome of children treated with haematopoietic-stem cell transplantations from donors expressing the rare C77G variant of the PTPRC (CD45) gene. Br. J. Haematol..

[9] Rothrock C.R., House A.E., Lynch K.W. (2005). HnRNP L represses exon splicing via a regulated exonic splicing silencer. EMBO J..

[10] Oberdoerffer S., Moita L.F., Neems D., Freitas R.P., Hacohen N., Rao A. (2008). Regulation of CD45 alternative splicing by heterogeneous ribonucleoprotein, hnRNPLL. Science.

[11] Preussner M., Schreiner S., Hung L.H., Porstner M., Jack H.M., Benes V., Ratsch G., Bindereif A. (2012). HnRNP L and L-like cooperate in multiple-exon regulation of CD45 alternative splicing. Nucleic Acids Res..

[12] Shukla S., Oberdoerffer S. (2012). Co-transcriptional regulation of alternative pre-mRNA splicing. Biochim. Biophys. Acta.

[13] Marina R.J., Sturgill D., Bailly M.A., Thenoz M., Varma G., Prigge M.F., Nanan K.K., Shukla S., Haque N., Oberdoerffer S. (2016). TET-catalyzed oxidation of intragenic 5-methylcytosine regulates CTCF-dependent alternative splicing. EMBO J..

[14] Saldi T., Cortazar M.A., Sheridan R.M., Bentley D.L. (2016). Coupling of RNA Polymerase II Transcription Elongation with Pre-mRNA Splicing. J. Mol. Biol..

[15] Rong J., Yin J., Su Z. (2015). Natural antisense RNAs are involved in the regulation of CD45 expression in autoimmune diseases. Lupus.

[16] Kong L., Zhang Y., Ye Z.Q., Liu X.Q., Zhao S.Q., Wei L., Gao G. (2007). CPC: Assess the protein-coding potential of transcripts using sequence features and support vector machine. Nucleic Acids Res..

[17] Consortium G.T. (2013). The Genotype-Tissue Expression (GTEx) project. Nat. Genet..

[18] Bao L., Zhou M., Cui Y. (2008). CTCFBSDB: A CTCF-binding site database for characterization of vertebrate genomic insulators. Nucleic Acids Res..

[19] Ziebarth J.D., Bhattacharya A., Cui Y. (2013). CTCFBSDB 2.0: A database for CTCF-binding sites and genome organization. Nucleic Acids Res..

[20] Faghihi M.A., Wahlestedt C. (2009). Regulatory roles of natural antisense transcripts. Nat. Rev. Mol. Cell Biol..

[21] Khorkova O., Myers A.J., Hsiao J., Wahlestedt C. (2014). Natural antisense transcripts. Hum. Mol. Genet.

[22] Farnebo M., Bykov V.J., Wiman K.G. (2010). The p53 tumor suppressor: A master regulator of diverse cellular processes and therapeutic target in cancer. Biochem. Biophys. Res. Commun..

[23] Qu X., Alsager S., Zhuo Y., Shan B. (2019). HOX transcript antisense RNA (HOTAIR) in cancer. Cancer Lett..

[24] Acuna L.G., Barros M.J., Nunez P., Penaloza D., Montt F., Pedraza D., Crossley K., Gil F., Fuentes J.A., Calderon I.L. (2020). The cis-encoded antisense RNA IsrA from Salmonella Typhimurium represses the expression of STM0294.1n (iasE), an SOS-induced gene coding for an endoribonuclease activity. Biochem. Biophys. Res. Commun..

[25] Luco R.F., Allo M., Schor I.E., Kornblihtt A.R., Misteli T. (2011). Epigenetics in alternative pre-mRNA splicing. Cell.

[26] Rothrock C., Cannon B., Hahm B., Lynch K.W. (2003). A conserved signal-responsive sequence mediates activation-induced alternative splicing of CD45. Mol. Cell.

[27] Tong A., Nguyen J., Lynch K.W. (2005). Differential expression of CD45 isoforms is controlled by the combined activity of basal and inducible splicing-regulatory elements in each of the variable exons. J. Biol. Chem..

[28] Xu Y., Zhao W., Olson S.D., Prabhakara K.S., Zhou X. (2018). Alternative splicing links histone modifications to stem cell fate decision. Genome Biol..

[29] Rahhal R., Seto E. (2019). Emerging roles of histone modifications and HDACs in RNA splicing. Nucleic Acids Res..

[30] Tan M., Luo H., Lee S., Jin F., Yang J.S., Montellier E., Buchou T., Cheng Z., Rousseaux S., Rajagopal N. (2011). Identification of 67 histone marks and histone lysine crotonylation as a new type of histone modification. Cell.

[31] Zhou H.L., Pan Q., Tominaga K., Blencowe B.J., Pereira-Smith O.M., Misteli T. (2014). Regulation of alternative splicing by local histone modifications: Potential roles for RNA-guided mechanisms. Nucleic Acids Res..

[32] Sorenson M.R., Jha D.K., Ucles S.A., Flood D.M., Strahl B.D., Stevens S.W., Kress T.L. (2016). Histone H3K36 methylation regulates pre-mRNA splicing in Saccharomyces cerevisiae. RNA Biol..

[33] Davie J.R., Xu W., Delcuve G.P. (2016). Histone H3K4 trimethylation: Dynamic interplay with pre-mRNA splicing. Biochem. Cell Biol..

[34] Yuan H., Li N., Fu D., Ren J., Hui J., Peng J., Liu Y., Qiu T., Jiang M., Pan Q. (2017). Histone methyltransferase SETD2 modulates alternative splicing to inhibit intestinal tumorigenesis. J. Clin. Investig..

[35] Pradeepa M.M., Sutherland H.G., Ule J., Grimes G.R., Bickmore W.A. (2012). Psip1/Ledgf p52 binds methylated histone H3K36 and splicing factors and contributes to the regulation of alternative splicing. PLoS Genet..

[36] Vakoc C.R., Mandat S.A., Olenchock B.A., Blobel G.A. (2005). Histone H3 lysine 9 methylation and HP1gamma are associated with transcription elongation through mammalian chromatin. Mol. Cell.

[37] Saint-Andre V., Batsche E., Rachez C., Muchardt C. (2011). Histone H3 lysine 9 trimethylation and HP1gamma favor inclusion of alternative exons. Nat. Struct. Mol. Biol..

[38] Bieberstein N.I., Kozakova E., Huranova M., Thakur P.K., Krchnakova Z., Krausova M., Carrillo Oesterreich F., Stanek D. (2016). TALE-directed local modulation of H3K9 methylation shapes exon recognition. Sci. Rep..

[39] Neems D.S., Garza-Gongora A.G., Smith E.D., Kosak S.T. (2016). Topologically associated domains enriched for lineage-specific genes reveal expression-dependent nuclear topologies during myogenesis. Proc. Natl. Acad. Sci. USA.

[40] Gorkin D.U., Qiu Y., Hu M., Fletez-Brant K., Liu T., Schmitt A.D., Noor A., Chiou J., Gaulton K.J., Sebat J. (2019). Common DNA sequence variation influences 3-dimensional conformation of the human genome. Genome Biol..

[41] Schmidt F., Kern F., Schulz M.H. (2020). Integrative prediction of gene expression with chromatin accessibility and conformation data. Epigenetics Chromatin.

[42] Ruiz-Velasco M., Kumar M., Lai M., Bhat P., Solis-Pinson A.B., Reyes A., Kleinsorg S., Noh K.M., Gibson T.J., Zaugg J.B. (2017). CTCF-Mediated Chromatin Loops between Promoter and Gene Body Regulate Alternative Splicing across Individuals. Cell Syst..

[43] Li Y., Haarhuis J.H.I., Sedeno Cacciatore A., Oldenkamp R., van Ruiten M.S., Willems L., Teunissen H., Muir K.W., de Wit E., Rowland B.D. (2020). The structural basis for cohesin-CTCF-anchored loops. Nature.

[44] Hashimoto H., Wang D., Horton J.R., Zhang X., Corces V.G., Cheng X. (2017). Structural Basis for the Versatile and Methylation-Dependent Binding of CTCF to DNA. Mol. Cell.

[45] Wiehle L., Thorn G.J., Raddatz G., Clarkson C.T., Rippe K., Lyko F., Breiling A., Teif V.B. (2019). DNA (de)methylation in embryonic stem cells controls CTCF-dependent chromatin boundaries. Genome Res..

